# Repetitive Transcranial Magnetic Stimulation Combined With Conventional Rehabilitation in a Patient With Lacunar Infarcts

**DOI:** 10.7759/cureus.99261

**Published:** 2025-12-15

**Authors:** Ayesha Juhi, Shreya Sharma, Dinesh Bhatia, Suman Dhaka, Rajesh Kumar, Deepak Kumar, Pritam Kumar Chaudhary, Pradosh Kumar Sarangi, Himel Mondal

**Affiliations:** 1 Physiology, All India Institute of Medical Sciences, Deoghar, Deoghar, IND; 2 Neuromodulation Laboratory, All India Institute of Medical Sciences, Deoghar, Deoghar, IND; 3 Biomedical Engineering, North-Eastern Hill University, Shillong, IND; 4 School of Liberal Arts and Center for Brain Science and Application, Indian Institute of Technology, Jodhpur, Jodhpur, IND; 5 Internal Medicine, All India Institute of Medical Sciences, Deoghar, Deoghar, IND; 6 Physical Medicine and Rehabilitation, All India Institute of Medical Sciences, Deoghar, Deoghar, IND; 7 Radiodiagnosis, All India Institute of Medical Sciences, Deoghar, Deoghar, IND

**Keywords:** case report, cognitive rehabilitation, ischemic stroke, lacunar infarct, motor recovery, neuroplasticity, physiotherapy, repetitive transcranial magnetic stimulation, rtms, small vessel disease

## Abstract

Lacunar infarcts, though small, can produce significant motor and cognitive impairments due to their disruption of cortico-subcortical networks. This case report describes a 45-year-old woman with subacute to chronic ischemic lacunar infarcts involving the right frontal white matter, right thalamus, and left lentiform nucleus, who presented with left-sided weakness and mild cognitive deficits. The patient underwent a six-week rehabilitation program combining repetitive transcranial magnetic stimulation (rTMS) with conventional physiotherapy. Excitatory 10 Hz rTMS was applied over the ipsilesional (right) primary motor cortex (M1) for 30 sessions across six weeks, with five sessions per week, at an intensity of 80% RMT. Functional outcomes were evaluated using the Fugl-Meyer Assessment (FMA) for motor recovery and the Montreal Cognitive Assessment (MoCA) for cognition at baseline, mid-intervention, post-intervention, and three-month follow-up. The patient showed steady improvements, with FMA scores increasing from 27 to 43 (out of 66) for the upper limb and from 15 to 24 (out of 34) for the lower limb, while MoCA scores improved from 17 to 27 (out of 30). The gains were maintained at follow-up, and no adverse effects occurred during treatment. These findings suggest that combining rTMS with physiotherapy may enhance recovery in patients with lacunar infarcts by promoting cortical reorganization and interhemispheric balance. This case highlights the feasibility and clinical relevance of rTMS-based neurorehabilitation, even in resource-limited settings.

## Introduction

Stroke continues to be a major disability-causing condition globally, with many survivors experiencing motor and cognitive problems that limit independence [1]. Among ischemic subtypes, lacunar infarcts (small, deep infarcts caused by occlusion of penetrating arterioles) commonly affect the frontal deep white matter, thalamus, and basal ganglia (e.g., lentiform nucleus) [2]. Even small lesions can result in significant impairments in motor control, processing speed, attention, and executive functions due to disruption of cortico-subcortical network connections [3].

The standard treatment for stroke patients focuses on controlling risk factors while performing exercises that target specific tasks [4]. Over the past decade, non-invasive brain stimulation (NIBS), including repetitive transcranial magnetic stimulation (rTMS), has been explored and is being studied as an adjunct to conventional therapy to modulate cortical excitability and promote network reorganization. rTMS works by delivering repeated magnetic pulses that modulate cortical excitability, which can strengthen neural pathways in the affected hemisphere and support functional reorganization after stroke [5]. Early studies suggest that carefully selected protocols may yield incremental improvements in upper- and lower-limb function and in cognition for some patients, but real-world data in small-vessel (lacunar) strokes remain limited [6].

This case report describes a 45-year-old woman with subacute to chronic ischemic lacunar infarcts involving the right frontal white matter, right thalamus, and left lentiform nucleus on CT. We document baseline motor and cognitive status using the Fugl-Meyer Assessment (FMA) for the upper and lower extremities [7] and the Montreal Cognitive Assessment (MoCA) [8], outline a 30-day rehabilitation program incorporating rTMS with conventional therapy and physiotherapy, and report short-term outcomes. The goal is to provide transparent, clinically applicable details that may inform individualized rehabilitation planning for patients with lacunar strokes.

## Case presentation

The patient was a 45-year-old female residing in a rural area of the Bardhaman district, West Bengal, India. She was an education-deprived homemaker who primarily spoke Bengali and Hindi. She had been diagnosed with hypertension in 2022, managed with amlodipine 5 mg once daily, and with diabetes mellitus (type 2) in January 2025. She had no prior history of stroke or cardiovascular disease. The patient lived with her husband, five children, daughter-in-law, and grandchildren in a joint family. There was no documented family history of stroke or cardiovascular disease. She avoided all substances, including tobacco and alcohol.

On the morning of March 7, 2025, she experienced sudden heaviness and numbness in her left arm and leg, followed within hours by progressive weakness and imbalance. Although speech remained intact, she noticed a slight slowing of her thinking. She was taken to a nearby hospital (primary health center) the same day, where initial clinical evaluation suggested a possible ischemic stroke. The treating physician advised an MRI of the brain, but the patient could not afford it at that time. Antihypertensive therapy and supportive measures were provided, after which she was discharged with residual left-sided weakness. The timeline of clinical events, assessments, and interventions is summarized in Table 1.

**Table 1:** Timeline of clinical events, assessments, and intervention milestones from stroke onset to three-month follow-up. rTMS: repetitive transcranial magnetic stimulation, AIIMS: All India Institute of Medical Sciences, FMA: Fugl-Meyer Assessment method, MoCA: Montreal Cognitive Assessment. References: Fugl-Meyer Assessment method [7] and Montreal Cognitive Assessment [8].

Date	Event
March 7, 2025	Stroke onset - sudden weakness in the left upper and lower limbs.
June 11, 2025	The patient was brought to AIIMS Deoghar for continued rehabilitation due to persistent motor and cognitive deficits.
June 12, 2025	Pre-intervention assessment (Motor and Cognitive Baseline: FMA and MoCA).
June 16, 2025	rTMS sessions initiated (10 Hz, 5 sessions/week, total 30 sessions over 6 weeks).
July 14 2025	Mid-intervention assessment (Week 4) for monitoring progress.
July 25, 2025	Intervention phase completed (30 sessions of rTMS + rehabilitation).
July 28, 2025	Post-intervention assessment (Motor and Cognitive).
October 25, 2025	Three-month follow-up assessment (sustained improvement in motor and cognitive domains).

**Figure 1:**
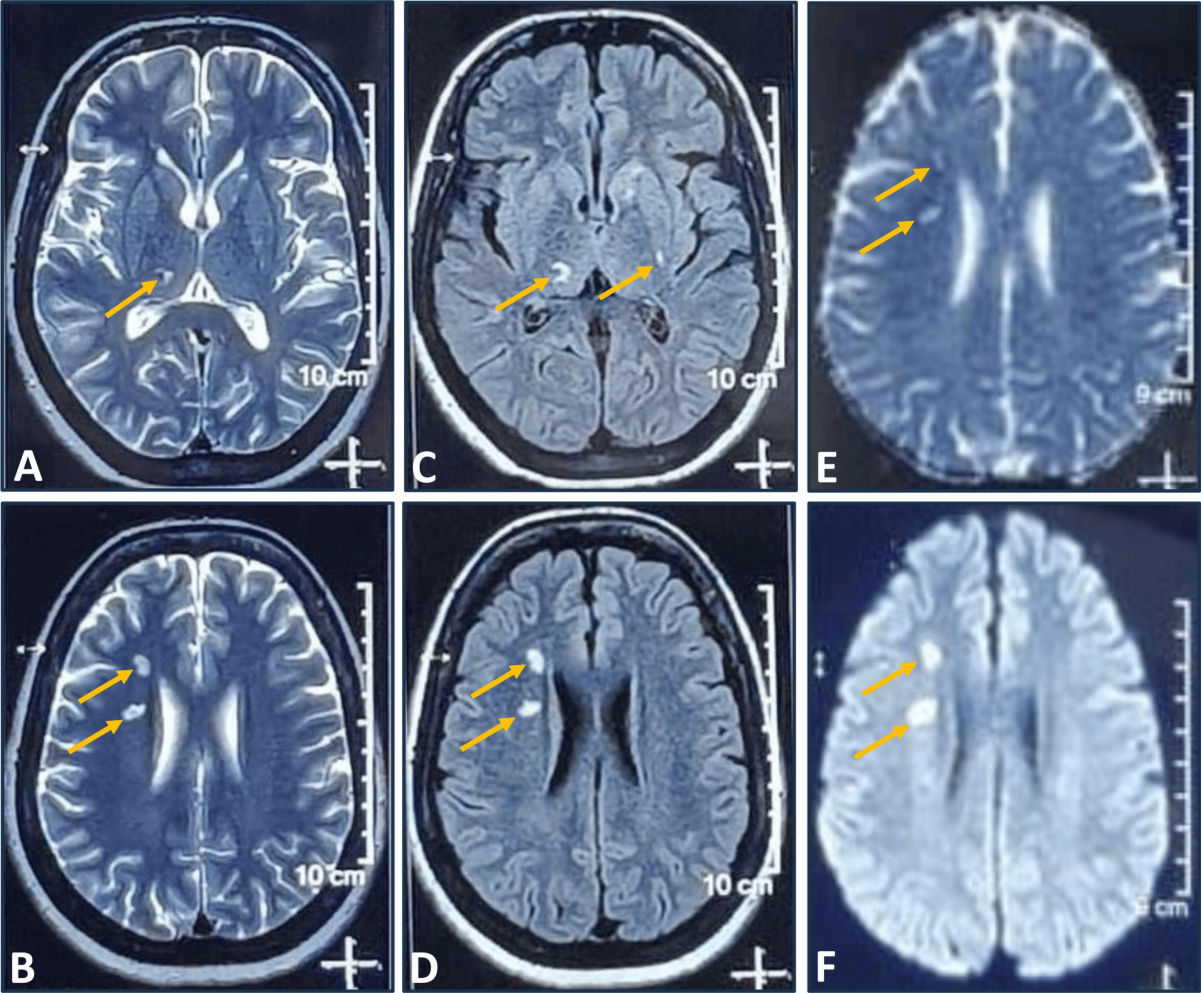
Axial MRI brain T2 (A, B) and FLAIR (C, D) images show a few foci of T2/FLAIR hyperintensities in the right frontal periventricular white matter, right thalamus, and left basal ganglia without restricted diffusion suggestive of old lacunar infarcts. The frontal lesions appear DWI hyperintense (E) without corresponding ADC hypointensity (F), suggestive of subacute–chronic infarcts. MRI: magnetic resonance imaging; T2: T2-weighted; FLAIR: fluid-attenuated inversion recovery; DWI: diffusion-weighted imaging; ADC: apparent diffusion coefficient.

On April 18, 2025, her MRI was performed, and the results supported the diagnosis of ischemic small-vessel disease. Axial MRI brain T2-weighted images (Figure 1a, 1b) and FLAIR images (Figure 1c, 1d) showed multiple punctate hyperintensities in the right frontal periventricular white matter, right thalamus, and left basal ganglia. The frontal lesions appeared DWI hyperintense (Figure 1e) without corresponding ADC hypointensity (Figure 1f), suggestive of subacute to chronic infarcts, while the remaining lesions represented old lacunar infarcts. These MRI findings and lesion locations confirmed that her left hemiparesis resulted from a subacute ischemic stroke affecting cortico-subcortical motor and cognitive networks.

As her weakness persisted and interfered with daily activities, she was referred to the All India Institute of Medical Sciences (AIIMS), Deoghar, Jharkhand, India, and arrived on June 11, 2025, for continued rehabilitation. On June 12, 2025, baseline clinical evaluation revealed that she was alert, oriented, and cooperative, with stable vital signs. Neurological examination showed left-sided hemiparesis involving both proximal and distal muscle groups, mild spasticity, and brisk reflexes on the affected side. Examination of the right side revealed normal tone, strength, and reflexes.

Baseline motor and cognitive assessments were conducted using the Fugl-Meyer Assessment (FMA) and the Montreal Cognitive Assessment (MoCA), respectively. At this stage, the patient scored 27 out of 66 for the upper limb and 15 out of 34 for the lower limb on the FMA, reflecting moderate motor impairment. Her MoCA score was 17 out of 30, indicating mild cognitive impairment, particularly in visuospatial, executive, and memory domains. Detailed domain-wise results across all assessment stages are provided in Table 2.

**Table 2:** Changes in motor and cognitive performance at baseline, mid-intervention, post-intervention, and three-month follow-up. UE: upper extremity, LE: lower extremity. References: Fugl-Meyer Assessment method [7] and Montreal Cognitive Assessment [8].

Assessment tool	Domain	Baseline (June 12, 2025)	Mid (July 14, 2025)	Post (July 28, 2025)	Follow-Up (October 25, 2025)
Motor function (assessed by Fugl-Meyer Assessment (FMA))	Shoulder/Elbow/Forearm (UE) (maximum score 36)	15	20	23	24
	Wrist (UE) (maximum score 10)	4	5	6	6
	Hand/Grasp (UE) (maximum score 14)	6	7	8	9
	Coordination/Speed (UE) (maximum score 6)	2	3	3	4
	Hip/Knee/Ankle Synergies (LE) (maximum score 28)	13	17	19	20
	Coordination/Speed (LE) (maximum score 6)	2	3	3	4
	Total UE (maximum score 66)	27	35	40	43
	Total LE (maximum score 34)	15	20	22	24
Cognitive function (Montreal Cognitive Assessment)	Visuospatial/Executive Function (maximum score 5)	2	3	4	4
	Naming (maximum score 3)	2	3	3	3
	Attention (maximum score 6)	4	4	5	5
	Language (maximum score 3)	2	2	3	3
	Abstraction (maximum score 2)	1	2	2	2
	Delayed Recall (maximum score 5)	3	4	4	4
	Orientation (maximum score 6)	3	4	5	6
	Total MoCA (maximum score 30)	17	22	26	27

**Figure 2:**
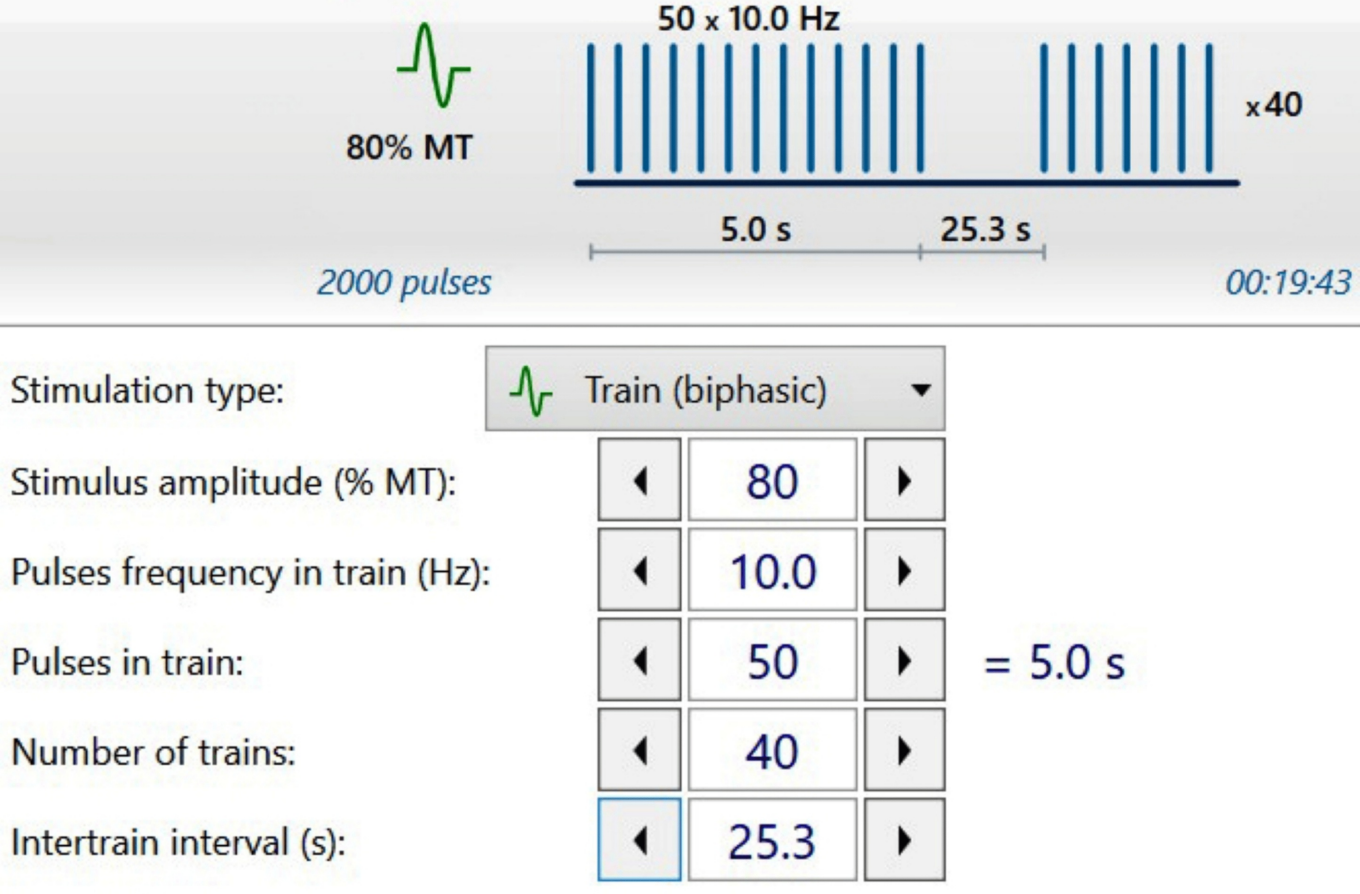
Repetitive Transcranial Magnetic Stimulation parameters, showing frequency, intensity, and session duration. MT: Motor threshold

The patient underwent a structured rehabilitation program integrating repetitive transcranial magnetic stimulation (rTMS) and physiotherapy. Treatment began on June 16, 2025, and continued for six weeks. rTMS was administered using a Repetitive Magnetic Stimulator (Model: Neuro-MS/D, Neurosoft LLC, Ivanovo, Russia). The protocol consisted of an excitatory 10 Hz frequency delivered over the ipsilesional (right) primary motor cortex (M1) at an intensity of 80% of her resting motor threshold. Each session lasted approximately 20 minutes and was conducted five times per week, totaling 30 sessions. The stimulation coil was positioned tangentially at a 45-degree angle to the midline over the motor hotspot. Alongside rTMS, the patient received daily physiotherapy that included task-oriented motor exercises, range-of-motion training, and gait retraining. She was also instructed to perform guided home-based stretching and fine motor exercises independently. The full stimulation and therapy parameters are detailed in Figure 2.

**Figure 3:**
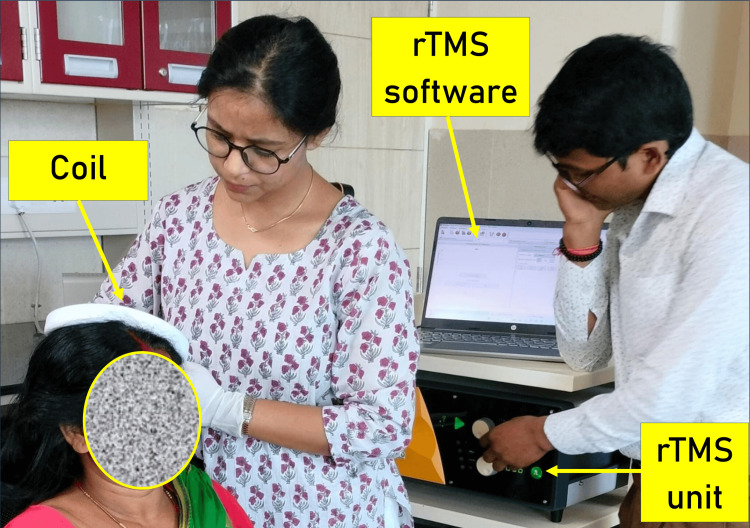
A patient receiving repetitive transcranial magnetic stimulation. rTMS: repetitive transcranial magnetic stimulation, Coil: coil that is used to provide stimulation.

A typical therapy session setting is shown in Figure 3.

By the mid-intervention stage on July 14, 2025 (four weeks), gradual improvements were observed in proximal arm and leg strength, smoother movement initiation, and better sitting balance. Upon completion of 30 sessions on July 25, 2025, her motor function had improved substantially, with greater control and coordination on the affected side. Post-intervention assessment on July 28, 2025, demonstrated significant motor gains, reflected in FMA scores of 40 out of 66 for the upper limb and 22 out of 34 for the lower limb. Cognitive function also improved, with a MoCA score of 26 out of 30, showing better attention and working memory.

At the three-month follow-up on October 25, 2025, her progress remained stable. The FMA scores reached 43 out of 66 for the upper limb and 24 out of 34 for the lower limb, indicating sustained recovery with improved coordination and strength. Her MoCA score increased to 27 out of 30, demonstrating better executive function, abstraction, and delayed recall. Functionally, she was able to walk independently with minimal support and perform daily activities with greater precision and confidence.

Throughout the intervention, no adverse events such as headache, seizure, or fatigue were reported. The patient tolerated rTMS sessions well and continued with home-based physiotherapy.

## Discussion

The case study follows a 45-year-old woman who developed subacute to chronic ischemic lacunar infarcts in the right frontal white matter, right thalamus, and left lentiform nucleus, which caused left hemiparesis and mild cognitive impairment. The small dimensions of lacunar lesions do not prevent them from damaging extensive cortico-subcortical networks, which produce both motor and cognitive symptoms. The treatment of such cases demonstrates how single vascular injuries in specific areas can produce multiple functional problems that require personalized rehabilitation strategies combining different treatment methods.

The treatment combined ipsilesional 10 Hz rTMS at the site of the stroke with physiotherapy, a protocol shown to enhance interhemispheric rebalancing and facilitate cortical excitability in the affected hemisphere. A single-pulse TMS test was used to determine the patient’s resting motor threshold before setting the stimulation power at 80% of her individual threshold. The treatment sessions took 20 minutes to complete, and therapy was given five times per week over six weeks. The treatment protocol focused specifically on modifying the M1 area [9].

The gradual but continuous improvements observed in Fugl-Meyer and MoCA scores over six weeks, with stable retention at three-month follow-up, support the feasibility of such combined interventions in small-vessel (lacunar) stroke cases.

Previous research has shown that rTMS treatment of motor cortex areas produces small yet effective improvements in motor function in stroke patients [10,11]. The current study provides relevant data for patients with lacunar infarcts, as these strokes are less frequently represented in NIBS research. The study contributes additional information through its evaluation of both motor and cognitive recovery at defined time points during the treatment period.

The observed recovery likely stems from improved interhemispheric inhibition balance and network reorganization across motor and cognitive domains. The 10 Hz rTMS delivered to the M1 site of stroke activated remaining corticospinal and corticothalamic circuits, enhancing signal transmission through intact neural pathways. The neuromodulatory effects of rTMS combined with task-specific practice likely enhanced synaptic strength and contributed to improved motor performance. The small but enduring cognitive gains in attention and executive control functions might result from fronto-motor network activation, which operates in areas that also support cognitive processes, thus indicating extensive adaptive changes beyond motor function. Although specific functional scales were not administered, the FMA domains broadly represent functional motor ability, and clinically, the patient had difficulty in walking independently, fine-hand tasks, and self-care.

This case presents three essential strengths through its use of proven assessment tools (Fugl-Meyer and MoCA) and its detailed treatment plan and scheduled follow-up appointments. The study faces three major drawbacks because it uses only one participant and lacks both sham control groups and neurophysiological evidence of cortical changes. The study faced two main limitations because it used delayed imaging to determine lesion age, and it lacked detailed MRC and Ashworth scoring data. The study demonstrates that rural rehabilitation centers with limited resources can achieve meaningful functional improvements through structured rTMS treatment combined with standard therapy for patients who have had a lacunar stroke.

Although early subacute initiation may be ideal, this case, treated in the late subacute to early chronic stage, demonstrates that neuromodulation treatment, when combined with cognitive and physical rehabilitation, can still produce clinically meaningful improvements and support more complete patient recovery.

## Conclusions

The patient showed continuous improvement in motor and cognitive functions, indicating that rTMS treatment with lesion-specific targeting and ongoing rehabilitation practices can improve stroke recovery in patients with lacunar stroke. Future studies should include larger participant groups and brain imaging studies to determine optimal treatment approaches and identify which patients are most likely to benefit from this therapy. In addition, the concurrent physiotherapy limits the ability to isolate the specific effects of rTMS.
